# Phenomenon-oriented lifeworld-led data generation in qualitative research-challenging absent or inadequate choices of methods

**DOI:** 10.1080/17482631.2026.2641162

**Published:** 2026-03-14

**Authors:** Helena Dahlberg, Christopher Holmberg, Karin Dahlberg, Elvira Pértega

**Affiliations:** a Institute of Health and Care Sciences, University of Gothenburg, Gothenburg, Sweden; b University of Technology Sydney, Sydney, Australia

**Keywords:** Qualitative research, lifeworld-led research, data generation, phenomenology, phenomenon-oriented research

## Abstract

**Purpose:**

This paper aims to articulate why and how the quality of data generation is important for developing valid qualitative research. The goal is to highlight how such an approach surpasses standardized or predetermined methods in generating high-quality data capable of illuminating existentially significant phenomena.

**Methods:**

We provide epistemological justification and methodological reasoning drawn from philosophy of science, and our extensive experience conducting and teaching qualitative research within healthcare contexts.

**Results:**

Appropriate data generation methods are found to be those inherently aligned with the nature and nuances of the phenomenon under study. We identify the importance of an initial thorough exploration of the phenomenon’s characteristics, ensuring methodological choices emerge organically from the research context itself. There is no method that answers all methodological questions. Instead, we emphasise that all methods have pros and cons.

**Discussion:**

Methodological decisions should not be dictated merely by convenience or convention within research groups. These decisions must embody sensitivity toward participants, particularly considering the vulnerable status of healthcare recipients. We advocate explicitly integrating human rights considerations into research practice, reinforcing ethical responsibilities inherent in qualitative health research. Adopting a lifeworld-led, phenomenon-oriented approach, represents a vital and ethically sound pathway to achieving insightful and meaningful qualitative research outcomes.

In qualitative research, as well as in quantitative research, focus has often been on the choice of analysis methods and their importance to high quality results. As a starting point, we argue that the choice of data generation methods is at least as important, even if they have not been recognised to the same extent. The quality of data is imperative when the aim is to analyse the data for meaning as in research focusing on existential matters, typically in phenomenological or hermeneutic approaches, or, for example, in ethnographic, anthropological, or grounded theory approaches, as well as in approaches aimed at thematic analysis. In this paper, the context of data generation is lifeworld-led research, but our ideas and conclusions are valid for all qualitative research.

A main background problem is that research in general is affected by a positivist stance to science, where there is one main and taken-for-granted approach, consisting of quantitative data and statistics. When researchers choose qualitative approaches, the givenness of approach and methods may remain. Without hesitation, the choice falls on an approach and a method one is used to and comfortable with, a method that advisors or mentors suggest, for example one that is common within one’s research group or institution. The competitive situation in contemporary research is also a factor that makes the process of planning an investigation too fast and maybe slovenly.

It may also be a positivist leftover to focus on language and words, which belong to the cognitive approach to science and are judged as more reliable than emotions and other existential matters that are not measurable and difficult to grasp. In such way, interviewing is chosen as a main method. We argue, that interviewing *may be* the best method in an investigation, but the choice must be based in the phenomenon in focus, not from somewhere else. There are studies in which methods other than interviews are better suited to the objects in focus. Such phenomena are embedded in culture and are characterised by tacit knowledge. It could also be a relational phenomenon, such as how people communicate through so-called double messages or through other forms of unclear messages. Klinke and Fernandez (2023) argue that “phenomenological interviewing is not, on its own, an adequate approach for studying certain objects, including those who cannot accurately report or describe their own experiences” (p. 172). Examples of this may be when participants with neurological psychiatric conditions cannot express themselves adequately or when there are hindrances to understanding. Further examples of when interviews are not accurate to use are with young children without language skills or persons with a foreign language, and there is no translator or other facilities to rely on.

In this paper, we aim to present epistemological and methodological arguments for the choice of data generation methods in qualitative research within the healthcare field. We draw on phenomenological philosophy of science, as well as our common extensive experience of qualitative research in the healthcare area, including teaching research on all levels. With the ambition to be practice-oriented, we use both published and other significant examples that demonstrate open and thoughtful choices of data generation methods. Additionally, the vulnerable position of persons in need of healthcare requires the integration of human rights considerations into general research practices, including data collection, which we also aim to address in this paper.

This is the second paper in a project on how to understand and describe rigorous qualitative research, which can be made accessible to students and researchers who are not well-versed in the philosophy of science or qualitative research. In a previous paper (Dahlberg et al., 2024), we advocated qualitative meaning analysis (QMA), which like this paper is based on a lifeworld theoretical approach. In that paper, we took a stand against the use of content analysis (QCA), which offers a seemingly pragmatic stance to healthcare inquiries but run the risk of adopting a mechanistic approach to qualitative analysis. We showed how this is problematic since it leads to low-evidence outcomes, and contend that the search for meaning, as in QMA, lies at the heart of qualitative enquiry in healthcare research dealing with existential phenomena such as health, illness, and care.

In this paper, to demonstrate the choice of data generation methods in qualitative research, we again oppose strategies that run the risk of adopting a mechanistic approach which in this case means advocating standardised or pre-selected methods for data generation. We critique against the hold on to methods one is convenient with or those that are common within one’s research group and preferred, time after time. Instead, we argue in favour of an open and rigorous phenomenon sensitive approach.

Below, we describe some characteristics of lifeworld theory and lifeworld-led research, which is our research context that functions as an exemplar and leads to the principal idea of being phenomenon oriented in the choice of methods for data generation in qualitative research. This is followed by a presentation of some qualitative methods and how to think through the choices and, not least, how to be aware of the pros as well as the cons of each method and mean in relation to the phenomena of study.

## Lifeworld theory and lifeworld-led research

Lifeworld theory corresponds to existential issues in the areas of health, illness, and care. Lifeworld-led research thus directs the attention towards the experiential field and everydayness Phenomenologically oriented research approaches usually build upon lifeworld theory and addresses experiential phenomena in, for example, pedagogy and education, psychology and psychotherapy, and health care (Ashworth, 2003; Bullington & Karlsson, 2008; Dahlberg, 2022; Dahlberg et al., 2024; Dahlberg & Dahlberg, 2019, 2020a, 2020b; Giorgi, 2009; Vagle, 2018; van Manen, 2016, 2025).

The description of the lifeworld varies, but it is mainly about selfhood, sociality, temporality, spatiality, embodiment, and emotional attunement (Ashworth & Chung, 2006; Klinke & Fernandez, 2023; Todres et al., 2007). These constituents, also called lifeworld fractions, can all be informative when attempting to understand the meaning of a phenomenon. Let us focus on “loneliness.” The researcher may then ponder whether loneliness is connected with time or place, how that experience may be bodily sensed or how it feels, and not least how it relates to other people. Such reflections can clarify the best way to move the data generation process forward.

Lifeworld-led research investigates how we relate to the world. Lifeworld is an idea and concept that describes the phenomenological attitude of not siding with any one position in dichotomies or dualisms. Instead, this approach focuses on that which is in-between, which relates the sides to each other, pointing to either similarities or discrepancies (Dahlberg & Dahlberg, 2026). For example, it is not about subjects *or* objects, but that which is in-between a subject and an object, and which relates a subject to an object. More precisely, the lifeworld describes our relationship with the world, i.e., how we experience our world, including objects and subjects, nature and culture.

Humans share the same world and social existence; we share language with others, as well as society, ethnicity, and e.g., customs. We share universal meanings such as the desire for meaningfulness, togetherness, love, and respect. At the same time, everyone perceives the world in one’s own personal wvanay, relating to one’s present situation, personal history and futural plans, one’s personal beliefs and values as well as one’s relationships to others. Although we share the same aspects of existence, we may experience them differently. The lifeworld differentiates us from and unites us with others. Uniqueness sets the tone of the way that *the how* of the sameness varies. Therefore, lifeworld theory is an essential approach to research that is sensitive to and respects every person and their agency (Todres et al., 2007).

The lifeworld is a key concept in phenomenological research and is of considerable importance in qualitative research in general, as well as in both data generation and data analysis. Lifeworld-led research investigates how we are always directed to our existential context, how we relate to others and ourselves, and what meanings arise within such relationships. An essential aspect of the lifeworld is our everyday life, where we sense, feel, think, dream, and so on. Merleau-Ponty’s way of describing the human perception points to the “business as usual” characteristics of everyday existence, as something that we feel so comfortable with but which at the same time can be seducing. In his Causeries for the French Radio, Merleau-Ponty (2004) addresses the lifeworld as the world of perception: “the world which is revealed to us by our senses and in everyday life.” This world, he says, “seems at first sight to be the one we know best of all. For we need neither to measure nor to calculate in order to gain access to this world and it would seem that we can fathom it simply by opening our eyes and getting on with our lives. Yet this is a delusion.” The lifeworld is “to a great extent, unknown territory as long as we remain in the practical or utilitarian attitude” (p. 39). Consequently, we must adopt an open research attitude to understand existential phenomena. Such attitude is important throughout an investigation, and not least when methods for data generation are chosen (Dahlberg, 2022; Dahlberg & Dahlberg, 2019, 2020a; Dahlberg et al., 2008).

## Phenomenon oriented data generation

Our alternative to standardised data generation methods is phenomenon-orientated choices. A phenomenon is something as it is perceived, experienced, or in any way lived through by someone. It can be a thing, an event, an illness, the care being given, or for example how illness and care influence daily life. Instead of beginning with a method on hand, lifeworld-led research is based on an open relationship to the phenomenon in focus (Dahlberg & Dahlberg, 2026). The choice of data generation method is led by how it seems to be, and what preliminary characteristics there may be at the beginning of an investigation. The aim to understand, describe, or explain a phenomenon and its meanings determines *what* method(s) should be practiced. The methodological guidelines originating from lifeworld theory state *how* methods are to be applied to obtain knowledge characterised by evidence.

In the initial phase of the research, an open phenomenon-oriented attitude means not being too quick in the choice of method(s), which is more difficult than one might think. Beginning in the research questions and the aim of the investigation, phenomenology and lifeworld theory contribute with essential epistemological and methodological principles, from which research can be planned in an evidence-based manner. As Gadamer (2004) describes, theory is something that can be really practical. Phenomenology and lifeworld theory do not function as governing theories; that is, they do not determine which method or methods should be used. Instead, following this line of thought, there is no one given “scientific” method.

Phenomenon orientation has several consequences, and there are thus issues to consider before data generation can begin. The choice of method is based on the awareness of how the participants’ experiential world may be illuminated in the best way and how the subjects are best approached for the phenomenon to be well explored. The choice of participants is one such. A central principle in qualitative research, particularly in a lifeworld-led approach, is that the amount of data or number of informants is less critical than the meaningfulness of the data. In this respect, there is the risk of restricting potential participants to narrowly defined profiles intended solely to generate data. Rather than relying on such preselected profiles, the lifeworld approach concentrates on how the phenomenon emerges in everyday experiences. For example, when investigating the phenomenon of “loneliness,” researchers should not seek a “lonely individual” per se, but rather examine “loneliness within the modern individual.” Thus, participants are chosen based on their relevant experiences and exposure to the phenomenon under study. Moreover, the quality of the data is influenced by contextual factors and, perhaps even more crucially, by the nature of the relationship between the researcher and participants. These considerations are explored in greater detail below.

The choice of data generation method must also consider human rights. As the Special Rapporteur on Cultural Rights (Xanthaki, 2024) cautions, without a human rights framework, scientific endeavours can be misdirected. Approaches that emphasise randomised controlled trials and meta-analyses risk neglecting service users lived experiences (Wieseler, 2016). For instance, the dominance of the biomedical model in psychiatry has often skewed evidence towards pharmaceutical solutions, contributing to increased coercion and unnecessary long-term use of pharmacy as well as severe side effects. Research requires diverse methods to ensure everyone involvement in shaping scientific progress and decision-making. In all research, including qualitative studies, there must be an awareness not to exclude traditionally marginalised population groups (Vagle, 2019).

On the other hand, there are qualitative approaches with the aim of public involvement that miss their goals designating research participants as “co-researchers” (Pértega et al., 2025). Such data generation strategy might obscure underlying power imbalances. Pertega et al. (2025) emphasise approaches that invite multiple perspectives to facilitate meaningful, equitable participation of “experts by experience”. In another recent article, Boyle and Stew (2025) explore the relevance of public involvement in research design and application. They conclude that phenomenological research may benefit from well-designed public involvement, and thus be regarded with greater credibility by professionals.

Lifeworld-led research together with a human rights-based approach to data generation are conscious resources to be allocated to ensure conditions are provided for meaningful engagement, as well as to ensure that participants’ priorities and perceptions are considered throughout the investigation (The Danish Institute for Human Rights, 2020). Both approaches also prioritise the inclusion of marginalised groups in research (OHCHR, 2018).

## Data generation methods

A study design can include more than one method, and all methods can be supported by different means. Interviewing is the most used method in qualitative research, followed by observations, which have been practiced for a long time in anthropology and ethnography (Dellenborg, 2020). This situation is not due to co-incidence but to their sensitivity to existential phenomena. In general, there is rich experience from these methods and how they are best practiced. As we show below, there is, however, much to think through when interviewing or observation become the main choice and, maybe more important, there are many more means to use in research. An ambition is to explore the pros as well as the cons of each method and mean.

## Interviewing

Due to the lifeworld focus, interviewing is a powerful method and very common in lifeworld-led and phenomenological research (Dahlberg et al., 2008; Kvale & Brinkmann, 2009; van Manen, 2016). In this section, we aim to thoroughly analyse and describe interviewing, including describing its essential characteristics, strengths and weaknesses. We also describe special interviewing such as on-line and in groups.

### Lifeworld-led dialogues

The practice of phenomenological, lifeworld-led interviewing can be understood as inspired by Merleau-Ponty (1995), who argues that there “is one particular cultural object which is destined to play a crucial role in the perception of other people: language” (p. 354). Many qualitative research studies have demonstrated how participants use colourful words to illustrate their own stories of lived experiences to illuminate a phenomenon of interest. Lifeworld-led interviewing allows participants to potentially formulate and make statements and descriptions that include all levels of experience, e.g., existential issues, deep emotions, arguments, ideas, opinions, and may reveal experiences that they had forgotten or had never previously thought of, or even experiences they have not previously been able to bring to the surface.

Albeit, to play such an essential role in research, there are several things to think through. First, one must consider if this is the very best method to reveal the meanings of the phenomenon in focus. Second, researchers must be aware of if/how the chosen participants have opportunity to express their experiences in order for the phenomenon to be illuminated. Third, it must be emphasised how the dialogue in a lifeworld-led and phenomenon-oriented interview is different from a typical everyday conversation between two persons.

Concerning the first and second critical instants, interviews may be used but together with corresponding methods or means (see below). Concerning the interview as a dialogue, the interviewer’s interest is to support the interviewees in illuminating their lived experiences of the study object. For the meaning of the phenomenon to be exposed, it is not enough to implement a day-to-day conversation, as if one is having a meeting with a neighbour. For example, traumatic experiences and memories and those that involve strong emotions of any kind can be difficult to convey even in research interviews (Jared, 2026). To this end, the interviewer must know how to support the participant’s ability of “coming into words” (Gendlin, 2004). The researcher’s stance during the interviews is of importance, being “the place of not knowing (…) making oneself vulnerable to discover (…) replacing judgement with curiosity” (Kiegelmann, 2009). With openness and genuine respect for the participant, what they know and experience, and for what they are contributing, the researcher communicates to the interviewee that s/he is worth listening to, and of great importance, in order to understand a phenomenon in focus. It is of further significance that interviewees are supported in using the expressions they need, thus creating the potential for new aspects of the phenomenon to be revealed. Besides openness and respect, spontaneity and flexibility are desirable interview characteristics that may increase the possibility of reaching beyond what has been previously accepted as factual, and beyond what has been taken for granted about the phenomenon. Lifeworld-oriented interviews are meant to be directed to individually lived experiences rather than to common opinions or to be part of the contemporary debate (Dahlberg et al., 2008; Kvale & Brinkmann, 2009; van Manen, 2016).

### Bodily awareness, immediacy, and presence

Since the researcher is a tool for collecting data, researchers can increase the potential for openness in interviews by learning how to implement bodily awareness, immediacy, and presence (Dahlberg, 2022; Dahlberg et al., 2008; Ferrándiz, 2011). An open attitude in the interview setting happens not only in one’s mind but also in the body. Merleau-Ponty (1968) highlights the importance of dialogical exchange that may characterise lifeworld-led interviewing: “A genuine conversation gives me access to thoughts that I did not know myself capable of, that I was not capable of, and sometimes I feel myself followed in a route unknown to myself which my words, cast back by the other, are in the process of tracing out for me” (p. 13). The quote shows the texture of a good dialogue when not only thoughts and words are shared but also one’s lifeworld, and an essential aspect of the lifeworld is the lived body, including bodily memories.

The researcher must engage in bodily awareness that supports immediacy and presence in the interview. It will not work if the researcher is busy thinking about what to ask later on, or how to analyse some interesting statements. Running an interview, one must have the ability to be here and now, and more sensing than thinking about one’s bodily posture, for example, if one’s shoulders are tight due to unease and stress, it may send stressful signals to the interviewee. Ongoing attentiveness and keying into what is occurring in our own bodies as a response to what the interviewees convey in words, phrases, and non-verbal actions may allow us to formulate additional relevant questions (Dahlberg, 2020; Vagle, 2018).

An interviewee’s posture is also communicating meaning, for example, whether it seems open or closed, relaxed or tensed, happy, or unhappy. The interviewees may be encouraged to focus on the feelings, bodily expressions, or bodily sensations that the researcher notices are evoked during the interview. Embodied emotions manifest in gestures and may be recognised as physical responses in participants’ bodies (e.g., “her legs shake when describing the horrible event”). Strong feelings or sensations expressed by interviewees may be noticed by the researcher, but they can also explore them together.

Researchers benefit from immediate and improvisational open awareness throughout the research process, but not least in interviewing (or other data generation activities). Being immersed in the research, one does not know what will show up the next moment, but must be attentive and ready for it (Dahlberg, 2022). In such openness, all the researcher’s senses are sharpened while simultaneously maintaining stillness; there is both an active and passive stance where one is ready to genuinely listen and be surprised. From this, we may conclude that, even if the interviewer is leading the interview, s/he should not try to dominate the dialogue. Both the researcher and the participant are following a route staked out by the phenomenon, a rout previously unknown to both.

### Sequences of an interview

Phenomenological interviews pend between active, directing, and passive follow-up questions or suggestions. The interviewer frames the interview with a few directing questions, preferably sufficient to be memorised. One special question is the initial question, which has the purpose of opening up the dialogue in a way that helps the participant feel comfortable, and at the same time directs attentiveness toward the lived, everyday experience. In a study on loneliness, the opening up was read: *Please, tell me about what an ordinary day look like!* (Dahlberg, 2007).

During the interview, the interviewer could use further questions that guide the participant to certain areas of interest that relate to the phenomenon, for example, those guided by intersubjective, temporal, spatial, or situational perspectives.

### In the loneliness study such questions or suggestions were: tell me about a time you felt lonely. Do you remember a particular event?

It is also relevant to include insights gained from previous interviews in this kind of interview sequence. In such a way, the interviewee may be gently urged to reflect upon something that would otherwise not come up in the interview.

The other kind of sequence in lifeworld-oriented interviews means asking many follow-up questions, such as “Can you give me an example?”, “can you tell me another time when ….?”, and to ask the interviewee to “tell me more.” There cannot be too much emphasis on the importance of these questions or prompts. They are essential because they support lifeworld focus and avoid both taken-for-granted experiences and shallower opinions. Such persistent asking, which might feel unpolite and awkward in everyday situations, often works surprisingly well in research interviews. This is partly due to patients’, clients’, or, for example, students’ deep need to (finally) be listened to.

### Distant interviewing

In an increasingly digital world, conducting interviews remotely via telephone or video conferencing platforms has become common practice, often valued for its convenience and ability to transcend geographical barriers. From a lifeworld-led perspective, however, they reshape the shared dialogical space and challenge the researcher’s embodied presence—a cornerstone of phenomenological enquiry (Dahlberg, 2020).

The absence of physical co-presence can impede the subtle, non-verbal communication that enriches face-to-face encounters. Without physical co-presence, subtle non-verbal cues are muted: posture, gesture, and the room’s affective tone are harder to sense. Researchers using telephone interviews often note this as a key limitation (Mulyadi et al., 2022). This sensory limitation creates a potential barrier to achieving the deep, trusting connection necessary for participants to “come into words,” especially when discussing sensitive or traumatic experiences. The technological interface itself—a screen or a voice without a body—can create a sense of detachment, making it harder for both researcher and participant to achieve the state of genuine, spontaneous exchange that allows new meanings to emerge.

In a lifeworld-led study of co-occurring obesity and adverse childhood experiences for adult women, Jared (2026) found that using a cloud-based video-conferencing platform proved to be a viable, robust option for data gathering and new knowledge attainment. An explanation for this conclusion is that the “distance” that Zoom offered gave the participants a sense of freedom that allowed them to reveal difficult life experiences. In addition, it is possible that interviewees felt that they could divulge information about their ACEs and obesity to the interviewer—who served as a type of stranger they would not meet again—thus rendering what they disclosed as anonymous and “safe”. A last tentative explanation is that being at one’s home for the interviews may have also provided an added sense of security and protection.

Despite the challenges, distant interviewing is not inherently contradictory to a phenomenon-oriented approach, provided the researcher actively works to mitigate its limitations. It would be preferrable to see the person using video, rather than just using telephone. The researcher must make a conscious effort to create a space of focused presence, minimising distractions and verbally affirming their engagement to build rapport. It may also be beneficial to explicitly acknowledge the nature of the medium with the participant, opening a dialogue about how it might feel to share experiences this way. While distant interviewing presents specific obstacles to capturing lived experience, a methodologically aware researcher can still facilitate a connection and dialogue that illuminates the phenomenon in focus.

### Group interviewing

The last comment about interviewing concerns the aim of studying interaction and communication. As described below, this aim is commonly met through observation. Interviewing participants in groups may also be an option. Phenomenological focus groups appear to offer some benefits. First, they enrich the data by prompting participants to reflect on and share their experiences in a group with others, who relate to the same focus. Second, they allow for clarification and mutual understanding among participants and between participants and the researcher, as was noted in a study on women’s changed expectations for pregnancy after perinatal loss (Côté-Arsenault & Morrison-Beedy, 2001). In this context, every participant’s story must be heard, requiring skilful facilitation (Bradbury-Jones et al., 2009). Researchers must encourage quieter or more reserved participants to share their perspectives while tactfully managing those who tend to dominate the discussion to “appease”. Such a balanced approach fosters a richer and more nuanced exploration of the phenomenon in question.

## Observation

There are several reasons for choosing observation as the main method in a study, and they can be summed up by the interest in understanding a phenomenon that is more of lived body than of verbal language, as well as more interaction between persons than of individual experiences. Observations are often part of ethnological or anthropological research that describes many situations to learn from (Dellenborg, 2020). Within phenomenological research, observations are described by van Manen (2006) and Klinke and Fernandez (2023). Observation may be part of fieldwork, which allows the researcher to situate in relation to the participants and the environment, as a “work of learning from experience” (Kiegelmann, 2009). Thus, researchers can share events with participants who can be invited to further contribute in ways that may be previously unknown to the researcher. The degree to which the researcher participates in the field may vary from null, passive, moderate, or active level of involvement (Ferrándiz, 2011). In some situations, the observer may have opportunities to participate in the activities of those they are studying; however, in others, this might be difficult or impossible. Bogdan and Biklen (2003) refer to this range of possible roles from the active participant to the passive observer as the “participant/observer continuum”.

A common argument for observation is interest in the social world. In other words, it is about what happens between humans and between humans and the world, not least in nonverbal interaction.

In a phenomenological study with the title: *When caring and learning converge. Patients', students' and supervisors' experiences of encounters in a dedicated education unit within psychiatric* care (Andersson, 2015), participant observations of students and patients were included, together with interviews and diary narratives. The reason for choosing observations was that the researcher wanted to see what happened in the interactions between the students and patients in clinical encounters. He participated in both spontaneous and planned encounters for one week. The observations were followed up by individual interviews, with both students and patients, and according to the documentation these interviews showed to be easier to implement than other interviews in the same project.

Observation contributes information that may not have come through in interviews. The social world with its characteristics is a background against which individuals and particular phenomena can stand out as figures. In observations, researchers can see what is otherwise invisible and can put into words what is otherwise mute. Observation is not so much about passively looking; instead, it is about searching. Paraphrasing Santos Guerra (1999, p. 425), it is about training the eyes and the mind so that observation can help reveal the meaning of what has been seen.

Albeit it is the very same involvement in the social world that elucidates “the more” and brings “a surplus of meaning” to the research, that also can bring “too much” (Dahlberg, 2006). In observations, researchers face much more than what relates to the phenomenon in focus. Some information is highly relevant, while others are irrelevant. It can be difficult for researchers to sift out what will contribute to insights into the phenomenon and its meanings and what will not. Consequently, one must be aware of where one´s attention lies (Sá Cavalcantes Schuback, 2006).

Another problem that is different in observation and interviewing is the foundational need to “word” the phenomena and lived experiences. During participant observation, the researcher’s field notes are the data (Merriam, 2009). When researchers write down their observations, only they word what happens in the observation. Thus, there is a risk of falling into “epistemic ventriloquism” (Haraway, 2019), that is, to narrow the phenomenon within a theoretical pre-establishment and to describe the “otherhood” based on epistemic inequality. This could be a possible pitfall. To avoid this, the study will benefit from adding formal interviews or talking to the people involved in the observation. Thus, some forms of original wording support the analysis (Dahlberg, 2006).

## Written accounts

As van Manen (2002) demonstates, writing “is not just externalising internal knowledge, rather it is the very act of making contact with the things of our world”. Textual expression has a mparticular value for phenomenological research.

For students and novice researchers learning qualitative research, written accounts may be the least complicated method to use. In phenomenological research, written accounts have been frequently used by Giorgi (2009) and his followers. This method is well suited to the form of analysis recommended by Giorgi, which means dividing the full text into meaning units and including every sentence in the analysis. Such detailed work is, for the most part, not possible, for example, if data consist of 15 interviews and around 45 minutes per interview, which ends up being several hundred pages of text.

However, because of one-sided communication, it is not a useful tool if the phenomenon of interest is multifaceted or complex in other ways. In such studies, it is important to have the opportunity to ask follow-up questions, not falling into the trap of ending up having only some taken för granted opinions as a result. The more complex a phenomenon is, the more complex the methodological approach must be to illuminate both meaning structure and nuances (Svanström & Dahlberg, 2004).

If the method of written narratives is chosen, the instructions to the participant are imperative: to write as concretely as possible about the phenomenon of interest, for example, what actually happened or what was seen or heard, etc., and to avoid summaries or far-going interpretations. Sometimes, it is constructive to ask for critical incidents or to encourage the participant to describe both the positive and negative experiences of that which is in focus.

In an educational study on the joy of learning, Cronqvist (2021) asked nine-year-old and twelve-year-old boys and girls to describe situations in which they felt joy in schoolwork. They were also asked to describe situations in which they were sad. This choice of method resulted in rich descriptions of students’ time in school and how joy proved to be an important component of learning.

## Methods and means that support interviews and observations

Research may be directed towards persons who by one or other cognitive or communicative disorder cannot easily communicate their experiences. For phenomena to be thoroughly understood and described, researchers may have to consider the capacity of the participant and what supportive means are required. Researchers need to overcome the centrality of words, which is limited when the phenomenon is ineffable (Laplantine, 2020). Alternative methodologies that displace words in the margins are required to address silence. Laplantine further suggests the use of multisensorial methods that are creative enough to explore alternative ways of expressing a surpassing language. This would be the case of studying forms of waking as a “pre-expressive behaviour” that is part of everyday life as expression of the “body-mind” (p. 10).

There are several means to support interviews and observations and strengthen data, as well as the following analysis. Recording interviews and conversations in observational studies makes transcription possible, which is important for thorough meaning-oriented analysis. Situations that occur in certain contexts, such as classrooms or inpatient care of special interest, can also be filmed.

### Art-based research

Arts-based research employs symbolism, metaphors, and artistic processes to explore the experiential domain and includes various forms such as visual (e.g., photography), narrative (e.g., prose and poetry), and performative (e.g., drama) methods. It addresses the limitations of traditional qualitative research by moving beyond the idea that knowledge can be expressed solely through language. Arts-based approaches facilitate non-verbal storytelling and multimodal expression, capturing experiences that are difficult to quantify, such as the subjective, relational, social, and emotional aspects of life. The field of art-based research is dynamic and expanding (Andersson et al., 2020; Blumenfeld-Jones, 2016; Larsson et al., 2013; Visse et al., 2019). Drawings and paintings can be important means of significant use if the participants are children or adults with verbal difficulties. Ready-made cards with different motives can serve this purpose.

### Body mapping

Body mapping is a specific arts-based method that aligns with embodied enquiry and recognises the interconnection of the mind and body. Defined by Gastaldo et al. (2012) it involves “creating body maps using drawing, painting, or other art-based techniques to visually represent aspects of people’s lives, their bodies, and the world they live in”. The process includes tracing a life-sized outline of the body and filling it with words, colours, and symbols, offering a holistic view of physical, emotional, and social experiences. Originally introduced to study physical health, body mapping has since been standardised in art therapy. It is frequently employed to explore mental health, social identities shaped by mental illness, minority identity, and traumatic events (Murray et al., 2023). Unlike many other arts-based methods, body mapping sees the body as a primary site for making meaning. Additionally, body mapping has proven to be effective in research with younger populations, as it facilitates diverse expression and creates a non-stigmatising environment.

### Photos

Participant-produced photographs were used in a project to describe how women with stress-related illness experience well-being in everyday life (Hörberg et al., 2020; Sternudd et al., 2024). Data were collected through lifeworld-oriented interviews based on photos, taken by the women, relating to well-being in everyday life. A typical photo had a balanced composition, depicted a closed space with isolated objects situated close to the beholder, and was taken from above. By portraying calm and manageable spaces, the photos visually suggested that qualities such as balance and control are important aspects of experiencing well-being. The findings show that surroundings and supportive environments are important for unconditional beingness, which supports well-being in everyday life. From another study in the same project, Gunnarsson et al. (2021) conclude that for women living with a stress-related disorder, a focus on well-being in everyday life might contribute a valuable complement to stress rehabilitation for occupational therapists and other health professionals. The authors also conclude that the use of photographs as a basis for reflections on everyday life and health/well-being was positive in the clinical context. In addition to combining interviews and photographs, this project used both quantitative and qualitative methods, and it included both patients/clients and therapists (Gunnarsson et al., 2021, 2022).

Olausson et al. (2013, 2014) investigated the meaning of being cared for or of being a nurse caring for patients in an intensive care unit, photovoice combined with interviews was used. In the 2013 study, either patients or the main researcher photographically documented the bed area from the patients' point of view. They were advised to capture the patient's room and objects that were emotionally meaningful to them. In the 2014 study, nurses were asked to photograph the bedspace and capture what was important to them in providing care. The photographs were used in the interviews.

### Drama

A powerful way to support both interviewing and observation is the use of drama, where a partly forgotten situation can be recalled in an embodied way. The participant is asked to play the roles of persons who were engaged in a focused event. Enacting an experience that may have happened long ago has the power to awaken memories and support vivid descriptions (Carlsson et al., 2002). After the drama session, the participants are asked to reflect upon the enacted event and can then contribute with further experiences and maybe more clarity of the phenomenon.

### Written accounts

Besides being the main choice of method, written accounts can also support interviews and observations. For example, let us say that a study is about a complex phenomenon, and it is difficult to plan the interview and directing questions. The interviewees can then be asked to submit a written account of the phenomenon a few days before the interview, which informs the interviewer of some preliminary viewpoints with which to go further in the interview. In a study on the lived experience of elderly with severe Alzheimer’s disease, the researcher asked the healthy partners to keep a diary for some weeks, which was informative for further planning of the project and the interviews (Svanström & Dahlberg, 2004).

### Sample size

Sample size is often an issue in qualitative research. A positivist leftover is the emphasis on a large number of participants, that is, the more data, the better. In quantitative research, a sufficiently high amount of data directly influences the statistical analyses and, consequently, the level of evidence. This is not relevant to qualitative investigations, such as lifeworld-led research, in which the question of meaning is essential (Van Wijngaarden et al., 2017). In order for evidence to characterise the outcomes, the data must be rich in meaning and variation. Important criteria concern sufficient depth in data, that is, they go beyond general opinions or other shallow expressions of current views.

On the other hand, the number of interviews, written accounts, and observations is not arbitrary. In a way, it is fair to say that the more data there are, the more likely it is to include enough meaning and depth. However, the problem is that too many interview transcripts, narratives, or observation protocols lead to such a large amount of data that it becomes too difficult to overview or include in meaning-oriented analysis.

Compared to quantitative research, qualitative research is more of a kind of handicraft. Instead of numbers, it centres on how human existence (in the end) is perceived and communicated through language and words (Dahlberg, 2022). Evidence comes from how well the methods match these phenomena. The sample size is related to such epistemological assumptions.

### Examples of contextual awareness

Phenomenology and lifeworld theory are sometimes (wrongly) accused of dealing with individual experiences and of excluding social and historical structures. As phenomenology is not part of naturalistic philosophies that take the world as it is for granted, but rather aims to discover *how* it comes to be the way it is, such research is concerned with unearthing the structures or conditions of possibility that constitute the world as we know it. Here, knowledge from all areas of expertise may serve well in discovering such structures.

In research, we occasionally encounter phenomena that warrant special attention. As Vagle (2019) suggests, certain phenomena should be approached as though they are continually formed and re-formed by a multitude of actants and forces, both human and non-human. He further notes that this perspective has long been present in the theorising and epistemologies of black, indigenous, and people of colour (BIPOC). Recognising this, researchers, particularly those from privileged white backgrounds, must broaden their perspectives and think more creatively about what constitutes data. According to Vagle (2018), in phenomenological research, we need to know how to find other kinds of material that can be explored to see how they might provoke our understanding and see a phenomenon differently. We must consider, for example, histories of oppression; the people’s own narratives and discourses; their spaces, places, contexts, and policies; and philosophical concepts, all of which can serve as phenomenological material and complement data from their own lived experiences. A particularly useful research approach to explore the experiences of interlocking oppressions is the participant observation technique of “shadowing”. Shadowing is a qualitative research technique conducted on a small scale, in which the researcher acts as an observer. In shadowing, researchers observe real-life situations of a research participant for a set period of time, being able to witness experiences of systematic discrimination on a daily basis, which provides an opportunity to see how different forms of oppression shape quotidian interactions with the world.

Such reasoning is also valid in situations where the phenomenon includes vulnerability; for example, in the areas of health care, psychology, psychotherapy, or education, when, for example, therapists or other professionals want to understand their patients’ or clients’ needs for help, teachers want to understand their students’ learning challenges, or when social workers want to understand poverty. These areas have in common that there are recipients of professional services in vulnerable situations, depending on others to get well, or to be able to live well. Seeking and receiving help is a sign of reliance, and such dependency must be considered when reflecting on patients’, clients’, or students’ roles and possibilities in meeting institutions and their professionals.

Another example of a contextual matter is gender awareness. Beauvoir (1989) and Young (2005) speak about the body as gendered and how it is seen and judged by others. When the phenomenological understanding of the body means *I can*, a gendered understanding illuminates how, in women, there instead manifests, and *I cannot.* To address the gendered dimension, the methodological feminist formula “the personal is political” allows exploring the social archaeology of the phenomenon in personal textures, making use of the biographies to speak about the social world (Lahire, 2004, pp. 37–47).

## Summing up

Qualitative enquiry focusing on meaning is a powerful strategy for exploring, understanding, and explaining human existence. Adequately implemented, such an approach has the potential to illuminate existential suffering and innate health capacities in patients, develop person-centred care, and at the same time ensure that the investigation succeeds in presenting evidence-based results. To perform such research, high-quality data must be obtained to reveal the meaning of existentially characterised phenomena.

The choice of data generation method in qualitative research cannot be arbitrary or habitual. To ensure high-quality and meaningful data, researchers must adopt a phenomenon-sensitive approach that prioritises lived experiences, participant needs, context and methodological openness. The more complex the phenomenon in focus is, the more thoroughly the methods must be estimated and chosen.

Lifeworld-led research is characterised by these qualities and can serve as an exemplar for qualitative research in general. Emphasising phenomenon orientation means that data generation methods cannot be standardised or chosen in advance. There are no fixed methods and no predetermined steps to be followed. The focus must be on the phenomenon, the object of research, and how individuals perceive the existential situations they encounter. Cognitive efforts are important but not enough; the lived body, including emotions, memories, hope, despair, happiness etcetera, must thus be involved in research. There must be methods of choice that have the capacity to relate to and illuminate also such meaning that is hidden or hard to hear. Every researcher must know how to invite the beginning of words as well as be open to be surprised.

## Ethical statement

This article did not include any personal data and consequently did not require ethical approval or informed consent to be granted.

## Data Availability

Not applicable.
